# Supplementary material for: The frequency, clinical characteristics and outcomes of Naja species related injuries in Malaysia consulted to Remote Envenomation Consultancy Services from 2020–2023
Source: PLoS Negl Trop Dis. 2025 Jul 7;19(7):e0013271. doi: 10.1371/journal.pntd.0013271 (PMC12258597; doi:10.1371/journal.pntd.0013271)
Supplement: S1 Table — (DOCX) [file pntd.0013271.s001.docx]

S1 Table. Association of length of hospital stay with type of envenomation and antivenom usage of *Naja* species bites cases consulted to RECS from 2020-2023

| Factors | Category | Total | Type of envenomation | | | χ^2^ | *p* |
| --- | --- | --- | --- | --- | --- | --- | --- |
|  |  |  | None (%) | Local (%) | Systemic (%) |  |  |
| Length of | <1 | 48 | 2 (4.2) | 45 (93.8) | 1 (2.1) | 63.929 | 0.000 |
| hospital stay | 1 | 153 | 7 (4.6) | 144 (94.1) | 2 (1.3) |  |  |
| (day) | 2-3 | 121 | 1 (0.8) | 103 (85.1) | 17 (14.0) |  |  |
|  | 4-5 | 33 | 0 (0) | 29 (87.9) | 4 (12.1) |  |  |
|  | 6-10 | 30 | 0 (0) | 20 (66.7) | 10 (33.3) |  |  |
|  | >10 | 11 | 0 (0) | 4 (36.4) | 7 (63.6) |  |  |

| Factors | Category | Total | Antivenom usage | | χ^2^ | *p* |
| --- | --- | --- | --- | --- | --- | --- |
|  |  |  | Yes  n (%) | No  n (%) |  |  |
| Length of | 0 | 48 | 1 (2.1) | 47 (97.9) | 119.952 | 0.000 |
| hospital stay | 1 | 152 | 6 (3.9) | 146 (96.1) |  |  |
| (day) | 2-3 | 120 | 48 (40) | 72 (60) |  |  |
|  | 4-5 | 33 | 14 (42.4) | 19 (57.6) |  |  |
|  | 6-10 | 29 | 19 (65.5) | 10 (34.5) |  |  |
|  | >10 | 11 | 8 (72.7) | 3 (27.3) |  |  |
