# Supplementary material for: The frequency, clinical characteristics and outcomes of Naja species related injuries in Malaysia consulted to Remote Envenomation Consultancy Services from 2020–2023
Source: PLoS Negl Trop Dis. 2025 Jul 7;19(7):e0013271. doi: 10.1371/journal.pntd.0013271 (PMC12258597; doi:10.1371/journal.pntd.0013271)
Supplement: S2 Table — (DOCX) [file pntd.0013271.s002.docx]

S2 Table. Comparison of antivenom usage and duration of hospitalization between the two *Naja* species cases

|  |  |  | Percentiles | | |  |  |  |  |  |
| --- | --- | --- | --- | --- | --- | --- | --- | --- | --- | --- |
|  | Snake species | N | 50th (Median) | 25th | 75th | IQR | Mean Rank | Sum of Ranks | Mann-Whitney U | P |
| Antivenom usage (Total amount in vials) | Naja kaouthia | 26 | 5.00 | 5.00 | 10.00 | 5.00 | 32.87 | 854.50 | 406.50 | 0.393 |
|  | Naja sumatrana (Bite) | 35 | 5.00 | 5.00 | 5.00 | 0.00 | 29.61 | 1036.50 |  |  |
| Length of hospital stay (Days) | Naja kaouthia | 96 | 1.00 | 1.00 | 2.75 | 1.75 | 111.75 | 10728.00 | 5640.00 | 0.615 |
|  | Naja sumatrana (Bite) | 122 | 1.00 | 1.00 | 2.00 | 1.00 | 107.73 | 13143.00 |  |  |
