# Supplementary material for: The frequency, clinical characteristics and outcomes of Naja species related injuries in Malaysia consulted to Remote Envenomation Consultancy Services from 2020–2023
Source: PLoS Negl Trop Dis. 2025 Jul 7;19(7):e0013271. doi: 10.1371/journal.pntd.0013271 (PMC12258597; doi:10.1371/journal.pntd.0013271)
Supplement: S3 Table — (DOCX) [file pntd.0013271.s003.docx]

S3 Table. Summary of mortality due to *Naja* species envenomation consulted to RECS from 2020-2023

| Demo-graphy | Date/time Incident | Date/time  Death | Incident Location | Case Details | Cause of Death | RECS Consult | Antivenom |
| --- | --- | --- | --- | --- | --- | --- | --- |
| Male,  33 y/o, Malaysian | 7/5/2023 @ 8.30am | 9/5/2023 @ 9.40am | Pengkalan Chepa, Kelantan | *N. kaouthia.* Bitten once at base of right thumb while cleaning a snake enclosure. Septic shock, respiratory failure. Intubated & given inotropes.  Cardiac arrest, unresponsive to CPR. | Septic shock (necrotizing fasciitis) | Yes  (post AV) | 15 vials (NKAV) |
| Male,  2 y/o, Malaysian | 30/1/2022 @ 5pm | 31/1/2022 @ 8.10am | Kuala Nerang, Kedah | Undifferentiated *N.* sp. Bitten once, at the right thigh while playing outdoor. Neurotoxic envenomation (altered mental status). | Respiratory failure. | Yes  (post death) | 4 vials (CRAV – inappropriate AV) |
| Male,  58 y/o, Malaysian | 20/1/2022 @ 8pm | 24/1/2022 @ 5.25pm | Ladang Linau, Sepang, Selangor | Undifferentiated *N.* sp. Bitten once on left foot while closing door of chicken coop. Vomiting & hypotension. Low GCS. Intubated & ventilated. CT scan, ischemic changes. | Basilar artery occlusion | Yes  (post AV) | 5 vials  (NPAV) |
| Male,  73 y/o, Malaysian | 21/1/2021 @ 4.30pm | Not specified | Sibu, Sarawak | Undifferentiated *N.* sp. Bitten once on right ring finger while picking up a chicken that fell into a drain. Neurotoxicity, CPR & intubated. CT scan showed cerebral oedema. AKI. | Hypoxic ischemic encephalopathy | Yes  (pre AV) | 10 vials (NKAV) |
