# Supplementary material for: The frequency, clinical characteristics and outcomes of Naja species related injuries in Malaysia consulted to Remote Envenomation Consultancy Services from 2020–2023
Source: PLoS Negl Trop Dis. 2025 Jul 7;19(7):e0013271. doi: 10.1371/journal.pntd.0013271 (PMC12258597; doi:10.1371/journal.pntd.0013271)
Supplement: S4 Table — (DOCX) [file pntd.0013271.s004.docx]

S4 Table. Comparison between the clinical pattern of injury, management, and outcome of *Naja kaouthia* and *Naja sumatrana* consulted to RECS from 2020-2023

| Factors | Category | Total | Snake species | | χ^2^ | *p* |
| --- | --- | --- | --- | --- | --- | --- |
|  |  |  | *Naja kaouthia* n (%) | *Naja sumatrana*  n (%) |  |  |
| Type of | Local envenomation | 238 | 107 (45) | 131 (55) | 0.63 | 0.729 |
| envenomation | Local and systemic envenomation | 21 | 11 (52.4) | 10 (47.6) |  |  |
|  | None | 8 | 3 (37.5) | 5 (62.5) |  |  |
| Local pain | No | 44 | 17 (38.6) | 27 (61.4) | 0.95 | 0.330 |
|  | Yes | 223 | 104 (46.6) | 119 (53.4) |  |  |
| Bite mark/ | No | 28 | 14 (50) | 14 (50) | 0.28 | 0.599 |
| puncture wound | Yes | 239 | 107 (44.8) | 132 (55.2) |  |  |
| Swelling | No | 97 | 43 (44.3) | 54 (55.7) | 0.06 | 0.806 |
|  | Yes | 170 | 78 (45.9) | 92 (54.1) |  |  |
| Dermonecrosis | No | 181 | 78 (43.1) | 103 (56.9) | 1.12 | 0.289 |
|  | Yes | 86 | 43 (50) | 43 (50) |  |  |
| Erythema | No | 209 | 96 (45.9) | 113 (54.1) | 0.15 | 0.702 |
|  | Yes | 58 | 25 (43.1) | 33 (56.9) |  |  |
| Local bleeding | No | 246 | 114 (46.3) | 132 (53.7) | 1.32 | 0.250 |
|  | Yes | 21 | 7 (33.3) | 14 (66.7) |  |  |
| Local numbness | No | 248 | 110 (44.4) | 138 (55.6) | 1.31 | 0.253 |
|  | Yes | 19 | 11 (57.9) | 8 (42.1) |  |  |
| Blister/bullae | No | 260 | 116 (44.6) | 144 (55.4) | 1.04 | 0.307 |
|  | Yes | 7 | 5 (71.4) | 2 (28.6) |  |  |
| Bruises | No | 264 | 120 (45.5) | 144 (54.5) | 0.00 | 1.000 |
|  | Yes | 3 | 1 (33.3) | 2 (66.7) |  |  |
| Tender enlarged | No | 263 | 121 (46) | 142 (54) | 1.76 | 0.184 |
| lymph node | Yes | 4 | 0 (0) | 4 (100) |  |  |
| Scratch mark | No | 265 | 120 (45.3) | 145 (54.7) | 0.00 | 1.000 |
|  | Yes | 2 | 1 (50) | 1 (50) |  |  |
| Vomiting | No | 244 | 113 (46.3) | 131 (53.7) | 1.13 | 0.288 |
|  | Yes | 23 | 8 (34.8) | 15 (65.2) |  |  |
| Ptosis | No | 257 | 115 (44.7) | 142 (55.3) | 0.90 | 0.342 |
|  | Yes | 10 | 6 (60) | 4 (40) |  |  |
| Altered mental | No | 265 | 119 (44.9) | 146 (55.1) | 2.43 | 0.119 |
| status | Yes | 2 | 2 (100) | 0 (0) |  |  |
| Drowsiness | No | 263 | 119 (45.2) | 144 (54.8) | 0.00 | 1.000 |
|  | Yes | 4 | 2 (50) | 2 (50) |  |  |
| Dizziness | No | 264 | 119 (45.1) | 145 (54.9) | 0.03 | 0.870 |
|  | Yes | 3 | 2 (66.7) | 1 (33.3) |  |  |
| Poor respiratory | No | 266 | 120 (45.1) | 146 (54.9) | 0.01 | 0.925 |
| effort | Yes | 1 | 1 (100) | 0 (0) |  |  |
| Paradoxical | No | 266 | 121 (45.5) | 145 (54.5) | 0.00 | 1.000 |
| respiration | Yes | 1 | 0 (0) | 1 (100) |  |  |
| Blurring of vision | No | 265 | 120 (45.3) | 145 (54.7) | 0.00 | 1.000 |
|  | Yes | 2 | 1 (50) | 1 (50) |  |  |
| Diplopia | No | 266 | 121 (45.5) | 145 (54.5) | 0.00 | 1.000 |
|  | Yes | 1 | 0 (0) | 1 (100) |  |  |
| Abdominal pain | No | 266 | 120 (45.1) | 146 (54.9) | 0.01 | 0.925 |
|  | Yes | 1 | 1 (100) | 0 (0) |  |  |
| Chest discomfort | No | 266 | 120 (45.1) | 146 (54.9) | 0.01 | 0.925 |
|  | Yes | 1 | 1 (100) | 0 (0) |  |  |
| Diaphoresis | No | 266 | 120 (45.1) | 146 (54.9) | 0.01 | 0.925 |
|  | Yes | 1 | 1 (100) | 0 (0) |  |  |
| Initial pain score | No pain | 44 | 21 (47.7) | 23 (52.3) | 4.54 | 0.337 |
|  | Mild | 76 | 39 (51.3) | 37 (48.7) |  |  |
|  | Moderate | 99 | 37 (37.4) | 62 (62.6) |  |  |
|  | Severe | 45 | 22 (48.9) | 23 (51.1) |  |  |
|  | Undocumented | 3 | 2 (66.7) | 1 (33.3) |  |  |
| Antivenom usage | No | 205 | 94 (45.9) | 111 (54.1) | 0.10 | 0.749 |
|  | Yes | 62 | 27 (43.5) | 35 (56.5) |  |  |
|  | NKAV | 53 | 25 (47.2) | 28 (52.8) |  |  |
|  | NPAV | 5 | 1 (20) | 4 (80) |  |  |
|  | Combination  (NKAV, NPAV) | 3 | 1 (33.3) | 2 (66.7) |  |  |
|  | Inappropriate antivenom  (HPAV, NKAV) | 1 | 0 (0) | 1 (100) |  |  |
| NKAV (vials) | 3 | 2 | 1 (50) | 1 (50) | 5.11 | 0.402 |
|  | 4 | 1 | 0 (0) | 1 (100) |  |  |
|  | 5 | 39 | 16 (41) | 23 (59) |  |  |
|  | 6 | 1 | 1 (100) | 0 (0) |  |  |
|  | 10 | 12 | 7 (58.3) | 5 (41.7) |  |  |
|  | 15 | 1 | 0 (0) | 1 (100) |  |  |
| NPAV (vials) | 5 | 6 | 2 (33.3) | 4 (66.7) | 0.89 | 0.346 |
|  | 10 | 2 | 0 (0) | 2 (100) |  |  |
| Wound | No | 250 | 109 (43.6) | 141 (56.4) | 3.65 | 0.056 |
| debridement | Yes | 17 | 12 (70.6) | 5 (29.4) |  |  |
| Skin grafting | No | 263 | 119 (45.2) | 144 (54.8) | 0.000 | 1.000 |
|  | Yes | 4 | 2 (50) | 2 (50) |  |  |
| Incision and | No | 265 | 119 (44.9) | 146 (55.1) | 0.72 | 0.397 |
| drainage | Yes | 2 | 2 (100) | 0 (0) |  |  |
| Intubation and | No | 263 | 119 (45.2) | 144 (54.8) | 0.462 | 0.497 |
| ventilation | Yes | 4 | 3 (75) | 1 (25) |  |  |
| Inotropes | No | 267 | 121 (45.3) | 146 (54.7) | 0.008 | 0.931 |
|  | Yes | 1 | 1 (100) | 0 |  |  |
| Length of hospital | 0 | 10 | 6 (60) | 4 (40) | 16.91 | 0.153 |
| stay (Days) | 1 | 109 | 44 (40.4) | 65 (59.6) |  |  |
|  | 2 | 50 | 22 (44) | 28 (56) |  |  |
|  | 3 | 17 | 9 (52.9) | 8 (47.1) |  |  |
|  | 4 | 8 | 3 (37.5) | 5 (62.5) |  |  |
|  | 5 | 7 | 3 (42.9) | 4 (57.1) |  |  |
|  | 6 | 3 | 0 (0) | 3 (100) |  |  |
|  | 7 | 7 | 6 (85.7) | 1 (14.3) |  |  |
|  | 8 | 4 | 3 (75) | 1 (25) |  |  |
|  | 14 | 1 | 0 (0) | 1 (100) |  |  |
|  | 15 | 1 | 0 (0) | 1 (100) |  |  |
|  | 18 | 1 | 0 (0) | 1 (100) |  |  |
|  | Not stated | 49 | 25 (51) | 24 (49) |  |  |
| Length of hospital | 0 day | 10 | 6 (60) | 4 (40) | 8.45 | 0.207 |
| stay (In range) | 1 day | 109 | 44 (40.4) | 65 (59.6) |  |  |
|  | 2-3 days | 67 | 31 (46.3) | 36 (53.7) |  |  |
|  | 4-5 days | 15 | 6 (40) | 9 (60) |  |  |
|  | 6-10 days | 14 | 9 (64.3) | 5 (35.7) |  |  |
|  | >10 days | 3 | 0 (0) | 3 (100) |  |  |
|  | Not stated | 49 | 25 (51) | 24 (49) |  |  |
| Morbidity? | None | 258 | 115 (44.6) | 143 (55.4) | 0.160 | 0.689 |
|  | Healed with scar | 9 | 6 (66.7) | 3 (33.3) |  |  |
| Mortality (Death) | No | 266 | 121 (45.5) | 145 (54.5) | 0.00 | 1.000 |
|  | Yes | 1 | 0 (0) | 1 (100) |  |  |
