# Supplementary material for: The frequency, clinical characteristics and outcomes of Naja species related injuries in Malaysia consulted to Remote Envenomation Consultancy Services from 2020–2023
Source: PLoS Negl Trop Dis. 2025 Jul 7;19(7):e0013271. doi: 10.1371/journal.pntd.0013271 (PMC12258597; doi:10.1371/journal.pntd.0013271)
Supplement: Attachment — Submitted filename: Response to reviewers.docx [file pntd.0013271.s005.docx]

**Response to reviewers:**


**Journal Requirements:**

1) We do not publish any copyright or trademark symbols that usually accompany proprietary names, eg ©,  ®, or TM  (e.g. next to drug or reagent names). Therefore please remove all instances of trademark/copyright symbols throughout the text, including:
- © on page: 8.
**Response:** The © attributed to the OpenStreetMap contributors has been replaced with “Copyright”. Base map static image from OpenStreetMap and OpenStreetMap Foundation. (Copyright OpenStreetMap contributors, https://www.openstreetmap.org/copyright).

2) Please upload all main figures as separate Figure files in .tif or .eps format. For more information about how to convert and format your figure files please see our guidelines: 
<https://journals.plos.org/plosntds/s/figures>
**Response:** The figures 1 to 3 were formatted using the Preflight Analysis and Conversion Engine (PACE) digital diagnostic tool.

3) We have noticed that you have uploaded Supporting Information files, but you have not included a list of legends. Please add a full list of legends for your Supporting Information files after the references list.
**Response:** the list of legends for the supporting information has been added after the references list.

4) Potential Copyright Issues:

- Please confirm (a) that you are the photographer of Figure 1, or (b) provide written permission from the photographer to publish the photo(s) under our CC BY 4.0 license.
**Response:** We have obtained content permission from the photographer M.K. Arif and indicated in the legend for each image in fig. 1.

- Figure 3. Please (a) provide a direct link to the base layer of the map (i.e., the country or region border shape) and ensure this is also included in the figure legend; and (b) provide a link to the terms of use / license information for the base layer image or shapefile. We cannot publish proprietary or copyrighted maps (e.g. Google Maps, Mapquest) and the terms of use for your map base layer must be compatible with our CC BY 4.0 license.
Note: if you created the map in a software program like R or ArcGIS, please locate and indicate the source of the basemap shapefile onto which data has been plotted.
If your map was obtained from a copyrighted source please amend the figure so that the base map used is from an openly available source. Alternatively, please provide explicit written permission from the copyright holder granting you the right to publish the material under our CC BY 4.0 license.
If you are unsure whether you can use a map or not, please do reach out and we will be able to help you. The following websites are good examples of where you can source open access or public domain maps:
* U.S. Geological Survey (USGS) - All maps are in the public domain. (http://www.usgs.gov)
* PlaniGlobe - All maps are published under a Creative Commons license so please cite u201cPlaniGlobe, http://www.planiglobe.com, CC BY 2.0u201d in the image credit after the caption. (http://www.planiglobe.com/?lang=enl)
* Natural Earth - All maps are public domain. (http://www.naturalearthdata.com/about/terms-of-use/).
**Response:** The direct link to the base layer outline of the map (Malaysia) is included in the Fig.3 caption “Base map static image and data from OpenStreetMap and OpenStreetMap Foundation. (Copyright OpenStreetMap contributors, <https://www.openstreetmap.org/#map=6/4.23/108.24)>.”

The copyright notice can be viewed at <https://www.openstreetmap.org/copyright>

Note: This same basemap has been used in past publications on PLOS NTD and its citation is the same.

**Reviewers' Comments:**

**Methods**

Reviewer #1: Objectives should be clarified.
Methods of transcribing the information from online text app in the form of consultancy and verification with case notes/case reports - need to be elaborated.
If the studies data were purely based on online text information, this should be made clear in the method description and discussed further.
**Response:** The RECS consultations data was extracted from each case that has been filed and kept at the Malaysian Bioinformation System (MyBIS) Toxinology. Not from transcription from online text app. This has been clarified in the methodology. Similar methods were utilized in publications using the same data source recently.
Ref:
1. <https://doi.org/10.1371/journal.pntd.0010983>
2. <https://doi.org/10.1371/journal.pntd.0010983>

Reviewer #2: Methods are well described but need some additional information.
Please include more overview of the RECS program either in the introduction or methods.
- For example: how do clinicians access the program? Do they have an app on their phone or via the computer?
**Response:** The details regarding the RECS consultation are explicitly explained in the introduction with the references to the RECS webpage as ref (10).
Ref: <https://toxed.com.my/recs>

- One suggestion I have is to provide more detail on inclusion and exclusion criteria of those in the study. On line 101 and 102 it mentions "alleged" cobra bite and unidentified snake-related injuries consulted to RECS were excluded. How was this determined? A simple flow map could work. For example, on line 104-106 it says the diagnosis was confirmed either by specimen brought to hospital or picture of actual specimen that caused injury or clinical presentation and syndromic approach. I think it is important to add more clarity to "clinical presentation and syndromic approach". What does that mean? Cobra is the focus of this investigation, but other venomous snake bites could be classified as cobra?
**Response:** As clearly mentioned in the methodology, only cases diagnosed by RECS consultants as *Naja* sp. related injuries are included in the study. This could either be due to confirmed specimen identification or from clinical features verified by RECS consultants. The categories of these were either *Naja kaouthia* bite, *Naja sumatrana* bite, Venom ophthalmia (by *N sumatrana*) and undifferentiated *Naja* sp bite (ie. the actual species were not available for specific species). The investigators do not make up the diagnosis based on the data analysis. This categorization of the diagnosis by RECS is as per the results.

Reviewer #3: The study objectives have clearly been articulated, and the study design is appropriate to address the study objectives. Sample selection and its number is also appropriate and commendable. However, it would be beneficial if the investigators could clearly describe the difference between an alleged cobra bite and an undifferentiated cobra bite. What is the rationale behind categorizing undifferentiated cobra bites? Please explain the case definition or the mechanism used.
**Response:** Thank you. There are two *Naja* species indigenous to Malaysia. An undifferentiated *Naja* sp bite reflects a cobra bite with pathognomonic signs and symptoms without the *Naja* sp being identified ie, no specimen or picture of the culprit cobra. These categories were made by RECS consultants during the consultation. It is part of the standard process/flow of the online consultation to have the diagnosis being verified by the experts in the field. We have included this in the methodology as part of clarifying the definition of terms of these diagnosis categories.

**Results**


-Does the analysis presented match the analysis plan?

-Are the results clearly and completely presented?

-Are the figures (Tables, Images) of sufficient quality for clarity?

Reviewer #1: See general comments below.

Reviewer #2: Results are reasonable. Tables and Figures are useful. Images of both N. sumatrana and N. Kaothia are meaningful for readers. I am very curious about "Undifferentiated Naja species bite (n=184, 36.0%)". It looks like the authors focused on the two Naja species present in Malaysia but it is unclear how the authors determined undifferentiated. Does this mean it was either sumatrana or kaothia? I would also like to see how the RECS consultants determined this and if any other "Cobra" bite occurred like those from King Cobra or possibly any outside Malaysia such as the Javan Spitting Cobra? Also were any of the envenomations due to non-native Cobra which were kept in captivity? Please comment
**Response:** As explained above, the diagnosis “Undifferentiated” *Naja* species bite refers to either *N kaouthia* or *N sumatrana* as per diagnosed by RECS consultants. This study does not include the diagnosis of non-native *Naja* species nor *Ophiophagus bungarus*. Unfortunately, the authors do not have the answer nor will make speculative statement to the question on how the experts made that diagnosis, but presumably it could be from many years of involvement in managing thousands of confirmed cobra bite envenoming in Malaysia. Though “Cobra” is also colloquially used for King Cobra, it certainly does not belong to the *Naja* genus. Based on the Malaysian MOH guidelines and several notable publications relevant to the region, there are differences in the behavior of these snakes, the mechanism of injury, presentation of the bite or strike, features of the bite itself, the development and progression of the local and systemic envenoming etc, Though the specimen of the snake may not be presented or the actual picture of the snake were absent, an intelligent guess to the offending culprit species between these two genus could be made. Certainly, there will be difficulties differentiating the *Naja sputatrix*, from the *N sumatrana* by untrained eyes and there is no telling if it is present in private keep in Malaysia. However, the specific geolocation of its occurrence alone would provide a safe distance from the assumption of its presence in the Peninsular Malaysia. Thus, emphasizing the importance of meticulous incident history documentation and close serial monitoring of the signs and symptoms progression generally called a syndromic approach.
Ref: <https://doi.org/10.1371/journal.pntd.0012359>

Reviewer #3: The results are understandable but need refinement.
Providing the nationality of snakebite victims is irrelevant, as snakebites depend on people's behavior, the snake’s behavior, and the environment, not nationality.
It's preferable to present only the most critical data instead of all findings, which may not be necessary for concluding the study. For example, occupations of victims, first aid methods, local envenoming signs, systemic envenoming signs, antibiotic usage, analgesic usage, features of acute adverse reactions to antivenom etc. (Tables 1, 2, and 3). Highlighting the most common presentations or findings is sufficient to understand the circumstances. Incorporating minor findings doesn’t influence the final picture. Or you may write them in the text without making the table too long. And too much lessor important information would deviate the attention from the most important findings.
**Response:** Thank you for the astute observation. However, the assumption that snakebite is not affected by Nationality in a multi racial and denominational Malaysia, which also relies heavily on foreign workers is somewhat speculative and inconclusive. This knowledge has great impact on the dynamics of the healthcare system and economics in Malaysia and how it can be improved. The authors decide to retain some of these “lesser important information” to reflect the completeness of the RECS documentation for each case consult. The three tables content has been trimmed.

**Conclusions**


-Are the conclusions supported by the data presented?

-Are the limitations of analysis clearly described?

-Do the authors discuss how these data can be helpful to advance our understanding of the topic under study?

-Is public health relevance addressed?

Reviewer #1: See general comments below.

Reviewer #2: Discussion and conclusions are sufficient. Authors did a great job of discussing their results between the N. sumatrana and N. kaothia envenomations. Limitations were also discussed. I would suggest authors add more in regard to undifferentiated Naja species bites since other "cobra" bites may have been involved in this study. This is mentioned in lines 204-207 but likely a limitation outside the scope of RECS program.
**Response:** Thank you. The concerns have been addressed.

Reviewer #3: The conclusion is well supported by the data, including a thorough description of limitations. However, the effect of a 12-hour delay in receiving antivenom may have impacted on complications and outcomes. This should be addressed more in the discussion section and conclusion. Finding a solution to reduce this delay should also be discussed in detail.
**Response:** Yes. As explained in the discussion, the delay in giving AV was due to the delay in transfer arrival from the initial district medical facility to a referral hospital. The specifics of the possible issues and what could be helpful were suggested. Finding a solution will be the recommendation for further studies.

**Editorial and Data Presentation Modifications?**


Use this section for editorial suggestions as well as relatively minor modifications of existing data that would enhance clarity. If the only modifications needed are minor and/or editorial, you may wish to recommend “Minor Revision” or “Accept”.

Reviewer #1: (No Response)

Reviewer #2: (No Response)

Reviewer #3: Minor revisions.

**Summary and General Comments**


Use this section to provide overall comments, discuss strengths/weaknesses of the study, novelty, significance, general execution and scholarship. You may also include additional comments for the author, including concerns about dual publication, research ethics, or publication ethics. If requesting major revision, please articulate the new experiments that are needed.

Reviewer #1: Authors reported a retrospective cohort study of confirmed Naja species cases consulted to Remote Envenomation Consultancy Services (RECS) from 2020 to 2023. The study aimed to identify the frequency, geographical distribution, clinical features, treatments, and outcomes of Naja species-related injuries, based on data transcribed from information in a smart text app which seemed to be the platform for the online consultancy. The finding is interesting and meaningful, especially for clinical management. However there are limitations which authors should consider to include in the manuscript, and more in-depth discussion should be made. Please see the comments per line numbers.

Line 90 - elaborate on subject matter experts (SME). What qualification does one need? Who were these experts and how the identification was verified?
**Response:** This has been addressed above with the ref link to RECS.

Line 96-97: Authors stated in the objective to the outcomes of Naja species-related injuries in Malaysia consulted to RECS. Were the consultations provided solely by RECS online without any other consultation/opinion given by other practitioners (specifically consultants within each hospital?) which could have influenced or in one way or another contributed to the treatment and outcomes. Please provide additional information at this point as to whether the treatment outcomes were fully results from consultation to RECS only without interference.

**Response:** As mentioned, RECS provides the appropriate advice to the healthcare professionals who manage the case. This consultation does not remove nor transfer the responsibility of the managing team to RECS.

Line 102-103: Data were extracted from "RECS consultation log and case records" - Please elaborate. What is the form of the RCES consultation log? Were these on smart text apps? How were these "record from smart texting app" transcribed into clinical data (log?), and how were these verified or cross-checked using the case records from hospitals? How many hospitals were involved and provided the primary data source which is important, how were the clinical case records reviewed and matched to the texts/data in the smart text apps?
**Response:** These were addressed in the earlier segments above.

Line 142: Query about undifferentiated Naja species... see comment below.

Line 159-160: Explain what were "undifferentiated Naja species"? Were these cases without the specimens and photos, or because of difficulty to identify? It might be better to consider stating it as "unidentified" cobra species?
**Response:** These were addressed in the earlier segments above.

Line 161: The commonest antivenom used was Thai Red Cross Naja kaouthia monospecific antivenom... (78.3%). Include more information regarding the remaining 22.7%? What antivenoms were given? How many did not receive antivenom?
**Response:** These were addressed in the Table 3.

Line 168-172: Provide details of complications: what complications specifically in the 38 (8.2%) patients? Of these, 34 were due to wound infection, what about the other 4? Please also provide details of time and severity of the complications/wound infection or local effect?
**Response:** These were addressed in Table 3. The time and severity are not documented nor able to be provided with this study objective.

Most frequent emergency resuscitation was for airway intubation and ventilation (n=21): Provide details, the time (onset), and the causative species and distribution?
**Response:** The time of onset were not documented nor able to be provided based on this study objective and data collected. The species were addressed in Table 3 “Systemic envenomation resuscitation”.

Line 180: Include comparative analysis for different antivenom usage? The majority were treated with the Thai Naja kaouthia monospecific antivenom and how about the others?
**Response:** These were addressed in Table 3.

Line 190: Four deaths were due to unidentified Naja species bite. What attempts had been to identify the species, did the patients received any treatment including ventilation and antivenom (what antivenom)? Any difference between "unidentified" and "undifferentiated" species?

**Response:** Refer S3 Table for details of the death cases. The term "undifferentiated" have been addressed above.

Line 196-197: It is crucial to state clearly whether this is a hospital based study or purely based on smart text apps-consultations. If this was the case (data fully derived from smart text apps on mobile phones), even though there were some statistical analysis of the "consultations" transcribed from the online consultation apps, authors should discuss the limitation especially for not able to capture cases not consulted to the RECS apps. Although the number of case was large, it is debatable how representative the finding is for the Naja bite in the whole Malaysia. It would be good to be more conservative in interpreting the results, which were unconventional to hospital-based epidemiological study and retrospective survey.
**Response:** These have been clearly mentioned in the introduction and methodology and limitations segments

Line 359-361: The difference in local envenoming severity....stress the need for species-specific management protocols. -This is true but in the result and explanation are not in-depth. Please provide more detailed comparison between the two, and if possible, representative images of the findings.
**Response:** the details requested are beyond the objective of this research.

Reviewer #2: Thank you for presenting your findings from the RECS program for two highly venomous cobra species found in Malaysia. The manuscript is written well, and data is important for ongoing surveillance practices of snake bite in this region of the world. Authors are experts in their field and have provided a well written manuscript.
**Response:** Thank you

In addition to the comments provided above I think it will be important to consider not generalizing the term "Cobra" in the title as there are additional species found in Malayasia and Borneo itself where cobra is used as a common name. Authors do not comment on "King Cobra", Ophiophagus hannah bites. Did any occur in this cohort? The Javan Spitting Cobra (Naja sputatrix) found in Borneo, albeit found in the southern regions of Indonesia on islands of Java and Lesser Sunda, is another species that may be involved in this cohort. Do we know if any included in this cohort were bitten by these species? Also, do we know if anyone had traveled from a region within Indonesia and back to Malaysia for care for cobra snake bite? My major recommendation is to ensure the RECS program is described well in the introduction and methods section. I would also recommend to the authors that they define "undifferentiated Naja species" better and whether any of these snakes could be non-native Naja from other regions of Asia or even King Cobra bites.
**Response:** The title has been modified. With regards to the *N. sputatrix* and other exotic nonnative *Naja* species, we did not encounter them in the diagnosis made by RECS. We however documented several *N, kaouthia* bite cases in the big cities of Sarawak (Sibu & Miri) which are hotspots for illegal wildlife and pet trade. A few bite cases from *N. kaouthia* were also documented in the cities of Johor Bahru and Melaka, the southern states of Peninsula Malaysia. Similar issue of concern with *Calloselasma rhodostoma*. Escapees from private collectors is also happening in Malaysia. The clinical burden is yet to be determined.
Related refs:
1. <http://dx.doi.org/10.1016/j.toxicon.2023.107023>

Reviewer #3: While many similar studies exist globally, studying geographically unique epidemiological data, clinical presentations, complications, and management of snakebites is crucial for improving patient outcomes. In this study, authors should focus more on presenting only the relevant data rather than including excessive information that makes the manuscript overly long.
**Response:** Agreed. Revisions have been made.
